# Renally Inappropriate Medications in the Old Population: Prevalence, Risk Factors, Adverse Outcomes, and Potential Interventions

**DOI:** 10.7759/cureus.49111

**Published:** 2023-11-20

**Authors:** Hamsa AlQashqri

**Affiliations:** 1 Community and Family Medicine, Umm Al-Qura University, Makkah, SAU

**Keywords:** renal functions, tools, interventions, adverse effects, risk factors, prevalence, old, medications, inappropriate, renally

## Abstract

Like most organs, the renal system decreases in function as we age. In the elderly, chronic kidney disease is common. When patients with chronic kidney disease take nephrotoxic medications, they are more likely to suffer adverse drug reactions, be hospitalized, and spend an extended period in the hospital. Calculating the renal clearance of a drug dose based on its glomerular filtration rate, or creatinine clearance, is necessary. Multiple tools are available for identifying renally inappropriate medications (RIMs). RIM prescriptions can be influenced by various factors, which vary according to the study. A higher number of medications means a higher likelihood of using RIMs. Numerous studies have investigated RIMs. The most contraindicated drug in renal insufficiency patients was a non-steroidal anti-inflammatory medication. A variety of interventions have been used to reduce RIM prescriptions to varying degrees of success.

## Introduction and background

The term chronic kidney disease (CKD) refers to damage to the kidneys or a reduction in glomerular filtration rate (GFR) that persists for more than three months [1].

As we get older, most of our organs do as well. For the kidneys, this is accompanied by intrarenal vascular changes; size and volume decrease with age as well [2]. Glomeruli decrease in number, and juxtamedullary nephron mass decreases. Consequently, the glomerular basement membrane's filtration area decreases, and its permeability decreases [2]. Aging results in a reduction in the glomerular filtration rate (GFR). As a result, they perform less well. As one ages after 40, the glomerular filtration rate (GFR) decreases by approximately 8 ml/min [2]. After the age of 65-70, this decline is more rapid [2]. As a result of age-related loss of renal mass and a decrease in the number and size of nephrons, chronic diseases such as diabetes and hypertension, and increased susceptibility to drug-induced damage, older adults are more susceptible to developing CKD [3]. According to statistics, kidney disease is an estimated 9.1% comorbid condition among older adults, with a prevalence range of 21.4 to 47.0% globally [3]. Although it is common and associated with mortality, CKD is often undiagnosed in older adults because serum creatinine, a by-product of muscle breakdown, is unreliable for predicting renal function. In older adults, reduced lean muscle mass often results in undetected renal insufficiency, even when serum creatinine levels are within the normal laboratory range [3]. As about half of all drugs or their metabolites are excreted by the kidneys, and 30% of all adverse effects of medication are either caused by or have a renal effect, it is important to determine if a patient suffers from renal insufficiency (CKD stages 2 through 5) [4].

Elderly patients are particularly susceptible to drug toxicity. Part of the reason is the lower excretion of drugs and metabolites from the kidneys, impaired by disease or aging [5]. A toxic insult can accelerate the decline of the glomerular filtration rate (GFR) in patients with chronic kidney disease. Additionally, chronic kidney disease patients who take nephrotoxic medications may experience adverse drug reactions, prolonged hospital stays, and an increased mortality rate [6]. Whenever renal insufficiency exists, renally excreted drugs should be reduced in doses, or time intervals between doses should be increased [5].

Internationally, the prevalence of seriously inappropriate medications prescribed varies between 19% and 80% [7-12]. Further variation exists based on the type of service provided: 13%, 34%, and 68%-80.5% in hospitals, long-term care facilities, and ambulatory settings, respectively [13].

There are few studies in Saudi Arabia that predict the prevalence of renally inappropriate medications (RIM). A retrospective study conducted at University Hospital found that 39% of older patients with renal impairment required dose adjustments [14].

## Review

Determining renal function

Measured creatinine clearance with a 24-hour urine collection provides the most accurate clinically available measurement of renal function. Unfortunately, this measurement is often difficult or impractical to obtain in the elderly [3].

Patients' serum creatinine levels may not reflect their renal function as they age [1]. Due to reduced muscle mass, a lower serum creatinine level does not reflect decreased renal function in elderly patients with CKD. For this reason, we should consider mild renal impairment when prescribing to the elderly [15]. The preferred method for estimating renal function (i.e., eGFR/1.73m2 versus eCrClr) and subsequently dosing renally cleared medications has recently been debated [3]. Equations for estimating the GFR improve on the accuracy of the levels of serum creatinine alone by incorporating demographic and clinical variables as surrogates for physiological determinants of serum creatinine [5].

Doses for drugs cleared renally should be calculated according to renal function (GFR or creatinine clearance). Cockcroft-Gault or Modification of Diet in Renal Disease (MDRD) equations should be used to estimate GFR, according to the Kidney Disease Outcomes Quality Initiative (KDOQI). Since 1973, clinical practice has relied on the Cockcroft-Gault equation despite studies showing that it underestimates GFR in the elderly [2]. There have been a few studies that have used Chronic Kidney Disease Epidemiology Collaboration (CKD‐EP) or Berlin Initiative Study (BIS1) equations [5,6]. MDRD and CKD-EPI equations were initially developed for epidemiological research and CKD staging, not for calculating dosages for patients with altered renal function; both equations significantly overestimate creatinine clearance in elderly individuals, resulting in incorrect dose calculations [4]. As a result, elderly adults should adjust their renal doses using the Cockcroft-Gault equation [7,10].

The term renally inappropriate prescriptions refers to drugs prescribed at the wrong therapeutic dose, based on a patient's GFR [5]. Chronic kidney disease medication management is complicated by multiple prescribers, complicated comorbidities, and underrepresentation in the literature [6]. Additionally, recommendations for the use and dosing of drugs in patients with renal impairment differed significantly between sources of information [7].

International tools/sources for identifying renally inappropriate medications

RIMs can be identified by several tools [3,8,9]. They were used in most RIM studies. An explicit method assesses potentially inappropriate prescribing by using predefined criteria based on evidence and expert consensus. Beer's criteria and "Screening Tool of Older Person's Prescriptions/Screening Tool to Alert Doctors to Right Treatment" (STOPP/START) [10] are among them. They are used to detect potentially inappropriate medications prescribed to elderly people that have been mis prescribed, overprescribed, or under prescribed [2].

In 1997, Beer's criteria were developed in the United States and updated in 2003, 2015, 2019, and 2023 by the American Geriatric Society. Beer's criteria were developed by Dr. Mark Beers to identify drugs whose risks outweigh their benefits. Among the most-consulted sources regarding the safety of older adults' medications is Beers Criteria. The AGS updated the Beers Criteria for potentially inappropriate medications in the elderly in 2015 by highlighting 20 drugs requiring dose adjustments based on kidney impairment. According to some studies, Beer's criteria can underestimate the prevalence of potentially inappropriate medications [10], and many of the drugs in Beer's criteria are rarely used in Western Europe, such as trimethobenzamide. Furthermore, the designation of some drugs as inappropriate by Beers’ criteria is questionable; e.g., amiodarone should be avoided regardless of diagnosis in older people [16]. As a result of their increased susceptibility to side effects and toxicities associated with long-term treatment, elderly patients are particularly at risk for complications. Although amiodarone may not often be a first-choice agent for controlling arrhythmias, it may be appropriate in certain instances [16]. Select patients at risk of ventricular arrhythmias or sudden cardiac death can safely take amiodarone shortly after acute myocardial infarction (AMI). In addition, amiodarone is effective for maintaining sinus rhythm after cardioversion from atrial fibrillation, but it should be used under the supervision of a cardiologist [17].

STOPP/START criteria were published in Ireland in 2008, and they were updated in 2015 and 2023 [9,18]. The use of physiological-system-based criteria facilitates their application because users can identify potentially inappropriate prescriptions by referring only to the appropriate physiological systems. Specifically designed for use in European countries, the STOPP/START criteria have shown high reliability among evaluators. STOPP criteria include drugs to avoid in older adults, drugs that increase the risk of falling, and drugs that interact with each other. Some drugs should be prescribed to older adults based on evidence of their effectiveness [2], according to the START criteria. It takes three minutes to apply the STOPP/START criteria. The STOPP/START criteria may serve as an effective and inexpensive tool for improving medication appropriateness in older patients once physicians become familiar with them [10]. STOPP/START criteria have been updated to minimize inappropriate prescribing in older people concerning renal function [2]. The latest version (Version 3) includes a section on 13 dosage problems (mainly renal) [19].

Contraindications and dosage recommendations still vary between screening tools. A beer's criteria state that 20 substances should be avoided or dose-reduced in people with renal impairment, but metformin is not listed. The Irish STOPP/START criteria only list six contraindicated drugs, including metformin. Under a glomerular filtration rate of 30 mL/min, this drug should be avoided. It varies significantly from official to official as to what the recommended dosage is for patients with renal impairment [8]. For criteria to remain useful, they should be regularly updated based on the ever-growing evidence-based literature on medication use in older adults [19].

In Saudi Arabia, we use both the AGS Beer Criteria and the STOPP/START Criteria [20,21]. There is a lack of widespread use of PIM detection tools in Saudi Arabia, according to the authors. It is, therefore, essential to develop educational programs to improve understanding of PIMs and facilitate the use of PIM screening tools in daily practice [21]. 

Table 1 shows the additional international sources for identifying renally inappropriate medications.

**Table 1:** Additional international sources for identifying renally inappropriate medications

Countries	Definition	Tools
Sweden, Denmark	Renbase provides a comprehensive knowledge base on drugs and renal function based on a comprehensive review of the published literature.	Renbase
Australia	Australian Medicines Handbook (AMH) provides drug information that facilitates safe, effective, and economical prescribing.	Australian Medicines Handbook (AMH)
Canada, Ireland	Informed decisions about medicines can be made at the point of care with information from the British National Formulary (BNF).	The British National Formulary (BNF)
France, Italy, Germany	Research and knowledge of a product's characteristics are used to prepare and update (SmPCs).	A summary of product characteristics (or SmPC)
Ireland	A valuable resource for nephrologists, specialists' nurses, and pharmacists treating patients with renal disease is the Renal Drug Handbook.	The Renal Drug Handbook
USA	Lexicomp system provides clinicians with evidence and recommendations to assist in treating and advising patients with complex medical conditions and histories, such as renal impairment.	Lexicomp

Prevalence

There have been a variety of reports on compliance with renal dosing guidelines, with values ranging from 19% to 80% [7-12]. Further variation exists based on the type of service provided: 13%, 34%, and 68%-80.5% in hospitals, long-term care facilities, and ambulatory settings, respectively [13]. A systematic review published in 2016 in Germany found that renally inappropriate drug use is prevalent in outpatient settings (1%-37%) and nursing homes (6%-43%) [2]. Nearly 72% of subjects with pre-dialysis chronic kidney disease were prescribed nephrotoxic medications in a retrospective cross-sectional study conducted in the US [12]. A retrospective observational study was conducted in Italian primary healthcare. The study found that 56% of medications were renally insufficient, suggesting renal function is not monitored, and inadequate awareness of CKD management is prevalent [22]. RIM may be more prevalent in primary-care settings because there are no reference texts for dosage adjustments. Also, laboratories do not calculate creatinine clearance when a creatinine test is requested, so clinicians must calculate it themselves [19]. As a result of underrecognizing renal impairment in primary care and failing to adjust medication dosages, RIM use was prevalent at admission [19].

The prevalence of potentially inappropriate medications in the Middle East is high in two studies conducted in Qatar (38.2%) and Lebanon (45.2%) [6,23]. Moreover, 58.4% of Kuwaiti patients received potentially inappropriate medication [24].

There is a variation in the prevalence of PIM prescribing among older Saudi Arabians. According to a study using 2003 Beers criteria, 43% of older adults used at least one potentially inappropriate medication, 18% used two potentially inappropriate medications, and 38.4% used three or more potentially inappropriate medications [9]. According to the second study, more than half of older patients who visited family medicine clinics or received home health care programs used one or more potentially inappropriate medications [10]. The prevalence of potentially inappropriate medications to be avoided was 57.6% among older adults in a large Saudi Arabian tertiary hospital [11]. Furthermore, 19% of geriatric cardiology patients had PIMs, according to a study [12].

There are few studies in Saudi Arabia that predict the prevalence of renally inappropriate medications (RIM). A retrospective study conducted at University Hospital found that 39% of older patients with renal impairment required dose adjustments [14]. There is a 15% overdosing rate of medications among patients undergoing hemodialysis in the outpatient unit [20].

It has been shown that RIM can be low, as shown in a study conducted in South Korea, where 5.3% of medication doses were excessive when considering renal function [9]. Further, a study conducted in the United States found that RIM was prevalent in 6% of elderly individuals [25], and a study conducted in Belgium found that 8% of elderly individuals received RIM [26]. There were only 1 out of 100 older adults living at home in Finland who used drugs with moderate-to-high nephrotoxicity risks [27].

The prevalence of RIM may vary between studies due to different inclusion and exclusion criteria, methods, and definitions [2]. It is also important to consider the equation for estimating renal function [2]. It is also possible that RIM prevalence may vary due to inconsistent implementation of dosage adjustment guidelines in cases of renal impairment. A recent study [7] found that familiar drug information sources define and classify renal impairment differently [28]. There are also a variety of ways to define inappropriate use of drugs, such as missed dose adjustments and contraindications [2,19]. A 2016 FDA warning label revision for metformin is an example. Metformin should not be started if the eGFR is between 30 and 45 ml/min. When the benefits and risks of metformin have been assessed, it can still be used, but it should not be used in patients with an eGFR below 30 ml/min. German guidelines changed the contraindication in 2015 to less than 45 ml/min eGFR [2].

Risk factors

Various risk factors can contribute to RIM prescription, and these factors may differ depending on the study. The study setting, the country, patient characteristics, and the tool used to detect RIMs may also affect the risk factors associated with the prescription of RIMs [29]. A greater probability of using renally inappropriate medications is found in patients being treated with more medications [8,9,12,13,15,19,30-37]. The potential explanations for this include the notion that the increased use of drugs increases the likelihood that some medications will have to be adjusted due to renal impairment [29,31,32,37].

Several studies have shown that aging is a risk factor for prescribing RIMs [12,15,34,38,39]. Age and renal impairment appear to be the two major pathophysiological factors not considered in drug dosing [39,40]. In contrast, young patients are more likely to take nephrotoxic medications, according to the Swedish/American study [41]. This may be because GPs prescribe fewer inappropriate medications to older patients due to their higher focus on renal function [32].

There is a well-established relationship between comorbidities and RIM prescriptions [2,11,18]. There is a greater risk of receiving inappropriate drugs when multiple diseases result in numerous prescribed medications [18]. It was more common to prescribe inappropriate medications to patients with hypertension [4,24,26], diabetes mellitus [21,24,25,27], hypercholesterolemia [26], atrial fibrillation, coronary artery disease, and heart failure [18,24,25]. Several drugs require dose adjustments when used by people with diabetes, hypertension, and dyslipidemia, which are also associated with chronic kidney disease [25,26]. Diabetes can also accelerate CKD progression, one of the most common diseases of the elderly [27]. In addition, CKD is associated with an increased prevalence of heart failure, contributing to CKD [27].

RIMs are also associated with advanced CKD [7,11,21]. Contraindicated prescriptions were more common in patients in the G4/G5 CKD stages than in stages G3a and G3b [12,21]. It may be because more drugs are classified as renal risks for patients with severely reduced renal function [30]. However, other studies have linked increased eGFR to a lower risk of RIM use [29,31]. It is more likely to require dosage adjustments when renal impairment is moderate to severe rather than mild. A physician may also prescribe these drugs to a healthy individual. Consequently, physicians should monitor prescriptions and renal function when eGFR values decrease [19].

62% of patients receive RIM prescriptions from primary care providers [16]. An Italian study found that diagnosing CKD by GPs did not reduce the likelihood of receiving prescriptions for nephrotoxic drugs. It appears that GPs are unaware of the dangers of prescribing inappropriate drugs for patients with CKD, which could result in their death [22]. Conversely, nephrologists' visits decrease RIM prescriptions. To improve disease management and reduce the risk of prescribing inappropriate drugs, GPs should seek nephrologist-specialist counseling from the earliest stages of CKD [33].

The results of some studies indicate that women are more likely to be exposed to RIMs [9,20,24], whereas the findings from other studies indicate that men are more likely to be exposed [25]. 

The most common renally inappropriate medication used

Different renally inappropriate medications have been investigated in different studies. Non-steroidal anti-inflammatory medications were among the most frequently contraindicated drugs in old patients with renal insufficiency [22,33,41], with acetylsalicylic acid being the most commonly inappropriately prescribed [22,35]. In addition to renal vasoconstriction and clinically significant reductions in glomerular filtration rate (GFR), NSAIDs cause nephrotoxic effects. Inhibiting renal prostaglandins or other mechanisms leads to interstitial nephritis, membrane glomerulonephritis, renal tubular acidosis, and papillary necrosis [42]. Low-dose acetylsalicylic acid is contraindicated in patients with severe CKD (GFR, 30 ml/min) [43] in addition. Patients with chronic kidney disease often have abnormal platelet function, making them susceptible to hemorrhage if treated with anticoagulants or antiplatelet agents [43]. On the other hand, in the early CKD stage there is an increase in cardiovascular risk for patients with CKD, and the benefits of aspirin may outweigh the risks of worsening kidney function [43].

According to a Swedish study, drugs that affect the renin-angiotensin-aldosterone system are commonly prescribed at excessive dosages, and antidiabetics are often prescribed when they are contraindicated [7]. As a result of efferent arteriolar vasodilation, ACEIs and ARBs reduce glomerular filtration rates [44]. The side effects of ACE inhibitors, such as hyperkalemia, metabolic acidosis, and the possibility of the further decline of the GFR, complicate their use in cases of advanced CKD [45]. On the other hand, studies showed that treatment with ACEI and ARB reduced the progression of CKD [46]. Physicians are aware of the arguments for and against ACE inhibitors in these patients but must compromise between the benefit and harm of this drug treatment [7]. Globally, diabetes is one of the leading causes of CKD [47]. A large proportion of CKD patients are being treated for diabetes, and antidiabetic drugs are excreted primarily by the kidneys, which may result in adverse effects [47]. The inappropriate use of antidiabetic medications increases the risk of hypoglycemia, cardiovascular disease, and lactic acidosis in CKD patients. The risk of lactic acidosis is higher with metformin in patients with advanced chronic kidney disease [31,48]. Nevertheless, lactic acidosis has been rarely associated with its use, and the guidelines are flexible regarding the mild to moderate stages of CKD [35].

Chronic kidney disease patients have a high incidence of hyperuricemia. As oxypurinol, the active metabolite of allopurinol, has a significantly longer elimination half-life in patients with kidney failure, allopurinol's dose should be adjusted according to eGFR. In general, the incidence of allopurinol-related AEs is low [49]. Allopurinol hypersensitivity syndrome is characterized by fever, rash, and internal organ involvement and is potentially life-threatening. However, there is evidence that allopurinol slows down the progression of renal disease in chronic kidney disease patients. Furthermore, allopurinol reduces cardiovascular and hospitalization risks in these subjects [49].

Fluid overload, hypertension, and heart failure are often symptoms of advanced CKD, which may explain why diuretics are prescribed [50]. Studies have shown that diuretics are frequently prescribed inappropriately [8,9]. Diuretics cause nephrotoxicity through hypovolemia, reducing plasma flow and resulting in acute kidney injury [50,51]. K-sparing drugs are well-known causes of RIM; however, as eGFR declines, caution is needed to reduce the risk of hyperkalemia and cardiovascular side effects [37].

In addition to the above-mentioned medications, proton-pump inhibitors are commonly prescribed inappropriately [9,31,34,52]. However, their indications for use are not always clear [53]. Taking PPIs for an extended period is concerning because of the increased risk of fractures, pneumonia, C. difficile infection, and, more recently, renal failure [54]. PPIs are prescribed frequently, suggesting clinicians don't deprescribe them after four weeks and after symptoms disappear [55]. 

Adverse outcomes

RIM can cause adverse effects, as is well known. According to a retrospective study conducted at a university hospital in Stockholm, Sweden, adverse drug reactions accounted for 14% of admissions, while impaired renal function accounted for one-third [56]. An Italian study of older patients with renal impairment found that RIM use was independently associated with a 46% increase in mortality [27] and a 40% increase in mortality in a French population-based study [36]. RIMs, however, were not associated with hospitalization or death in one study of community-dwelling older adults [24]. That can be due to increased provider monitoring or specific medications used in specific clinical situations not captured in a community-based cohort [33].

A retrospective cohort study in six community hospitals in the USA found that patients exposed to nephrotoxic drugs or cleared by the kidneys had serious adverse drug effects (51.1%) or significant adverse drug effects (44.4%). The most common ADE was worsened renal function. There were cases of bradycardia/hypotension and hypoglycemia in 3.7%, oversedation in 2.4%, and nausea in 1.2% [46]. An increased risk of falling can be another side effect of RIM [57].

A retrospective cohort study in Japan showed that exposure to two or more prescriptions of RIM accelerates the progression of CKD [58]. However, one recent retrospective cohort study did not show an association between RIMs and CKD progression. Presumably, this may have occurred because that study used end-stage renal disease or the composite endpoint of end-stage renal disease [57].

Additionally, nephrotoxic medication exposure increased healthcare utilization, including prescription drug use, emergency department visits, and hospitalizations. Furthermore, total health expenses were nearly 30% higher in nephrotoxic-medication-exposed groups than in non-exposed groups [13]. Healthcare costs have increased because subjects susceptible to nephrotoxic medications consume more prescription medications and medical care [12].

Interventions

Interventions have been carried out in a variety of ways. To reduce older people's use of RIMs, healthcare providers must collaborate in the treatment of older people with drugs [5].

Multidisciplinary rounds are less likely to prescribe RIMs than routine rounds in Japanese teaching hospitals and facilities [56]. By facilitating the sharing of information regarding ADEs in multidisciplinary rounds, physicians and other healthcare providers may be able to avoid prescribing RIMs [56].

In primary care, there is insufficient time to assess renal function and make appropriate recommendations [49]. Swedish primary healthcare researchers used a computerized decision support system (CDSS). This feedback system visualizes renal function in different degrees and prescribes and supports patients with guidelines for ensuring proper adherence to recommendations and ease of use [49]. According to the GPs, CDSS was fast, simple, and easy to use, and they wanted to continue using and recommending it. There was no examination of the effects of CDSS on RIM prescribing in this study [49]. In hospitals in America, computerized order-plus decision support systems improved dose appropriateness by 13% and frequency appropriateness by 24% [37]. While drug dosing appropriateness has improved, 49% of the applications' drug orders remain inappropriate, as physicians often ignore or override them. In some cases, overrides were appropriate due to low initial doses, such as prophylactic doses [59] or for patients who were critically ill [37]. Some may have ignored the advice due to their established practices [37]. Another hospital study found physician alerts to prompt physicians to choose and adjust drug dosages faster for patients with rising serum creatinine levels, reduced the risk of severe renal impairment by 55%, preserved renal function, and were well accepted by the physicians involved [60,61]. Moreover, Nash found that the baseline rate of excessive dosing in patients with renal impairment decreased from 23.6% to 17.3% after adding an automated system to an existing computerized physician order system [62]. Moreover, the cost of drug acquisition was reduced by $7,082 [63].

During a cluster-randomized controlled trial in a German primary care center, physicians were provided with interactive workshops on the detection and treatment of CKD, a desktop checklist of medications to avoid in CKD patients, information leaflets, and training on calculating creatinine clearance and dosage requirements. More than 30% of prescriptions over the standard daily dose were reduced, while over 70% of prescriptions over the maximum daily dose were reduced [45].

Pharmacists may also intervene by recommending that the doses, the intervals, or both in inadequate prescriptions be modified based on the degree of CKD. The prescribing physician recommended it verbally or in writing form [55]. Pharmacists are also vital in reducing RIM prescribing as members of the multidisciplinary comprehensive geriatric assessment (CGA) team. In the Netherlands, an observational study was conducted on the strategy to reduce medication errors in patients with renal impairment. First, automatic laboratory alerts were generated, then these alerts were linked to pharmacy data to judge the need for drug adjustments, and finally, pharmacists discussed the recommended changes with physicians [61]. Pharmacists' interventions resulted in decreased contraindications to drugs prescribed based on renal function (from 53.0% to 27.5%), reduced excess polypharmacy, and decreased preventable events from 14 to 5. In addition, pharmacist intervention saved approximately US $2,250 [59,64,65].

Healthcare workers, including general practitioners (GPs), pharmacists, and nephrologists, should work together to exchange relevant patient clinical information. The use of routinely collected data from electronic patient records, such as laboratory results about renal function, should be improved [55].

Deprescribing is another intervention that eliminates unnecessary and ineffective medications [66]. Identifying PIMs and prioritizing them for discontinuation or dose reduction when prescribing is essential. A plan for withdrawing the drugs safely and monitoring should follow these steps. This process should be accompanied by periodic kidney function evaluations [8]. It has been shown that this method improves patient satisfaction, decreases healthcare utilization, and lowers healthcare costs [67].

Additionally, a specific tool can enhance RIM effectiveness. The STOPP/START criteria were applied to chronic senior facility residents during a follow-up period, resulting in a reduction of 70.5% in PIM use and potential prescription omissions (PPOs). The intervention group also reduced falls and medication expenses significantly compared to the control group. Every month, 103 Israeli shekels (US$29) are saved per person [68].

In Saudi Arabi there is a lack of widespread use of potentially inappropriate medications detection tools, according to the author. It is, therefore, essential to develop educational programs to improve understanding of PIMs and facilitate the use of PIM screening tools in daily practice [21].

Hospitalization can also reduce RIM prescribing. In the period between admission and discharge, the use of PIMS decreased from 55% to 48% and 21.9% to 3% [3,23,27].

Complementary and alternative medicine (CAM)

Complementary and alternative medicine (CAM) is any healthcare approach that is not part of mainstream (i.e., Western or conventional) medicine. A complementary approach is one that uses a nonmainstream approach alongside conventional medicine; an alternative approach is one that uses a nonmainstream approach instead of conventional medicine [69]. As part of the complementary and alternative medicine group, herbal medicines are recognized [70].

As a result of the Dietary Supplement Health and Education Act of 1994, supplement manufacturers were allowed to sell supplements directly to consumers without having to submit proof of safety or efficacy to the US Food and Drug Administration (which classified supplements as a food category rather than a drug). These products are often marketed with inaccurate and possibly misleading information. Moreover, products rarely come in reliable or consistent potencies and dosages, making it difficult to conduct research on safety or efficacy [71]. Additionally, they are frequently processed or prepared incorrectly, with inadequate quality control, inadequate labeling, and insufficient patient information [70]. As a result of all the above factors, in addition to patients not informing their healthcare providers of natural product use, the toxicity profile of individual botanicals is often unknown [69].

As a part of the world, Saudi Arabian herbal products are mostly unregulated, with unknown ingredients, origins, or preparation methods. In most cases, these products are consumed without medical advice and lack scientific and evidence-based values, posing a severe risk to the consumer's life [70].

Although CAM is not regulated, it is widely used worldwide. Saudi Arabian adults use CAM between 65% and 80% [72,73]. The use of CAM in the US population ranged from 34% in one study to 62% in another study, respectively [74,75]. In addition, 49% of the French population, 46% of the German population, and 69% of the Australian population have used complementary and alternative medicine at some point in their lives (Fisher and Ward 1994; Xue, Zhang et al. 2007).

A nephrotoxic herbal medicine may cause kidney damage, but the exact incidence is unknown [76]. There is little information on the incidence of kidney injuries caused by Chinese herbal medicines [76]. Most of the information reviewed is derived from singular case reports, so causal relationships cannot be established [69].

Nephrotoxicity can be caused by several factors, including the intrinsic toxicity of herbs, improper processing or storage, adulteration, heavy metal contamination, incorrect dosage, and interactions between herbal medicines and medications [76]. In addition to acute kidney injury and chronic kidney disease, herbal medicine can also cause nephrolithiasis, rhabdomyolysis, Fanconi syndrome, and urothelial carcinoma in the kidneys [76]. 

Natural products may be particularly harmful to individuals with or at risk of renal dysfunction, either through renal complications associated with some natural products or through the accumulation of the natural product due to reduced renal clearance (Nauffal and Gabardi 2016). Individuals with concomitant medication use and older adults may be especially vulnerable. It has been shown that dietary supplements containing herbs that worsen glycemic control or improve blood pressure can indirectly lead to or worsen CKD (Grubbs et al., 2013). Additionally, dietary supplements containing herbs that increase diarrhea and vomiting can cause acute kidney injury, an established risk factor for CKD (Grubbs et al., 2013).

Aristolochic acid nephropathy, associated with misuse of some traditional Chinese herbal medicines, is the most reported herb-induced kidney injury (Chinese herbal nephropathy) [69,77]. It leads to rapidly progressive kidney interstitial fibrosis and urothelial carcinomas (Grubbs et al., 2013). In a retrospective study, aristolochic acid nephropathy was presented as CKD in 87% of cases and as AKI in 22% [76]. In addition to anthraquinones, flavonoids, and glycosides, the leaves of the creosate bush (Larrea tridentata), liquorice (Glycyrrhiza glabra), yohimbine, and willow bark (Salix daphnoides) are known to cause kidney toxicity [78].

Although many CAMs can cause nephrotoxicity, and their ingredients and mechanisms are quite complex, some necessary prevention measures should be employed to reduce or even avoid renal damage, including safe manufacturing processes that ensure high standards that avoid contamination with dangerous substances, as well as clear labels that indicate what compounds (and how much) are present in herbal medicine, licensing practitioners to ensure that they are knowledgeable of the potential side effects of traditional Chinese medicines, in addition to limiting the dose and duration of treatments as well as monitoring for adverse effects, and understanding the possible interactions between herbal medications and other medications [76].

## Conclusions

A person's renal function decreases as they age. Multiple comorbidities and multiple medications put older adults at risk for receiving RIM. RIM prescribing is common. Several medications can cause that, but NSAIDs are most blamed. The prescription of RIM may have adverse effects, as worsening renal function increases the risk of hospitalization and death. There are several interventions in healthcare systems designed to decrease the prescribing of RIM, one of which is the CDSS. Even though many tools and interventions are available to overcome the problem, further interventional studies are still needed to solve it.

In addition, CAM use is not regulated by the government. It is unclear how often a kidney injury occurs because of its use. The most common herb-induced kidney injury is Chinese herb nephropathy. Several preventative measures should be employed internationally and on a governmental level in order to reduce or even avoid renal damage.
